# Gene co-expression networks contributing to the expression of compensatory growth in metabolically active tissues in cattle

**DOI:** 10.1038/s41598-019-42608-w

**Published:** 2019-04-15

**Authors:** Kate Keogh, David A. Kenny, Sinead M. Waters

**Affiliations:** Animal and Bioscience Research Department, Animal and Grassland Research and Innovation Centre, Teagasc, Grange, Dunsany, Co, Meath, Ireland

## Abstract

Compensatory growth (CG) is an accelerated growth phenomenon which occurs in animals upon re-alimentation following a period of dietary restriction. The objective of this study was to perform gene co-expression analysis on metabolic tissues of animals undergoing CG, in order to elucidate the molecular control governing this phenomenon. Thirty Holstein Friesian bulls were fed a restricted diet for 125 days, after which they received feed *ad libitum*. Following 55 days of *ad libitum* feeding all animals were slaughtered. RNAseq and gene co-expression analyses were performed on tissue samples collected at slaughter including liver, rumen papillae and jejunum epithelium tissues. A period of CG resulted in 15 networks of co-expressed genes. One network of genes, involved in proteasome core complex, signal transduction and protein synthesis was found to be similar across liver and jejunum tissue datasets (r = 0.68, P = 0.04). Results from this study also showed that a large portion of co-expressed genes had not previously been implicated in the expression of CG, thus this study identifies novel genes involved in controlling CG across tissues, with hub genes holding potential for use as biomarkers for the selection of animals with a greater propensity to display CG.

## Introduction

In animal production systems, provision of feed represents the largest direct cost incurred by producers, accounting for up to 80% of the total costs of production^1^. Thus methods by which these costs may be reduced without hampering overall animal performance are of interest to the beef industry. One such method is the incorporation of compensatory or catch-up growth into production systems. Compensatory growth (CG) may be defined as a physiological process whereby an animal has the potential following a period of dietary restriction to undergo enhanced growth and efficiency upon re-alimentation, enabling it to achieve its pre-determined weight^2^. This naturally occurring phenomenon, which allows animals to cope with fluctuations in feed availability^3^, is widely used across animal production settings, particularly in ruminant production as a means to reduce feed-related costs^2,4^. This is facilitated through a reduction in overwintering feed costs in beef cattle^5^. However, although widely utilised, knowledge of the molecular mechanisms controlling CG are still lacking. A greater understanding of the biochemical control regulating CG in cattle would allow for greater utilisation and enhanced incorporation of this naturally occurring phenomenon into beef cattle production systems. In particular, knowledge of the key genes controlling CG would facilitate the identification of genetic biomarkers which following appropriate validation could be applied through genomic selection breeding programs to select animals with a greater propensity to display CG, ultimately improving profitability in beef production systems.

We have previously evaluated the molecular control of CG through the identification of differentially expressed genes across tissues including skeletal muscle, liver and ruminal and jejunal epithelium^6–9^. Results from these studies indicated that processes such as metabolism, cellular interactions, cellular growth and protein synthesis are contributing to CG. However, although these results provide information on tissue specific molecular mechanisms controlling CG, they do not explain the interactions between genes governing the expression of the trait. Indeed studies have shown that individual genes do not work alone, instead genes interact with each other to elicit a subsequent physiological phenotype^10–12^. Thus the identification of highly co-expressed genes that contribute to the expression of complex traits may reveal more information on the molecular control of a particular trait^13^. Moreover, the identification of hub genes, which are important in regulating the expression of several other genes within a network of co-expressed genes, may hold potential as biomarkers for the selection of a trait of interest^14^. We have previously undertaken a preliminary gene co-expression analysis to determine the contribution of jejunal epithelium to the expression of CG^15^. Results from this analysis identified co-expressed genes involved in functions including gene expression and translation as contributing to the expression of CG. The objective of the current study was to evaluate co-expression of genes during CG in organs closely associated with metabolism and digestion. Our study was focused on liver as well as ruminal and jejunal epithelium tissues which are involved in metabolic processes. Additionally, these tissues have repeatedly been shown to display accelerated growth upon re-alimentation following a prior dietary restriction, compensating ahead of other tissues or organs during the initial stages of re-alimentation^5,16–18^, thus, establishing the importance of these particular tissues to CG in cattle. The relationship between co-expressed genes and CG was further evaluated though determining the correlation between networks of co-expressed genes and factors contributing to CG. These factors included average daily gain (ADG), dry matter intake (DMI) and feed conversion ratio (FCR). During CG, cattle display greater growth rates apparent through greater ADG^2,4,5^; this is combined with an enhanced appetite evident through greater dietary intake or DMI^4,5^. Finally FCR, the ratio between DMI and ADG is used as a measure of feed efficiency and was included within this study as cattle undergoing CG display enhanced feed efficiency during re-alimentation following a prior period of dietary restriction^2,5^. The results presented in the current study build upon our previous preliminary analysis through the inclusion of additional datasets pertaining to varying metabolically active tissues. However the objective of the current study was to evaluate gene co-expression patterns of genes that were strictly commonly expressed across liver, ruminal and jejunal epithelium transcriptome datasets, this is in contrast to an evaluation of all expressed genes within a particular tissue type as previously presented for the jejunal epithelial transcriptome dataset^15^. Moreover we also sought to compare gene co-expression patterns across the varying tissue types which was undertaken through Weighted Gene Co-Expression Network Analysis (WGCNA) Consensus analysis^19^.

## Results

### Animal performance

Performance of animals undergoing CG is described in detail in Keogh *et al*.^5^. Tissue samples used in this study are derived from Holstein Friesian yearling bulls that were fed a restricted diet (RES treatment group) for 125 days (Period 1) and then subsequently fed *ad libitum* for a further 55 days (Period 2). A contemporary group of Holstein Friesian bulls (ADLIB treatment group), that received feed *ad libitum* for the duration of the 180 day trial was used to determine the magnitude of the CG response observed in RES animals^5^. Dietary restricted animals achieved their target growth rate during Period 1 growing at 0.6 kg/day, during the same time ADLIB animals grew at 1.9 kg/day. During re-alimentation induced CG in Period 2, RES animals displayed CG, gaining 2.5 kg/day with ADLIB animals gaining 1.4 kg/day during the same time. Overall, animals undergoing re-alimentation induced CG compensated for 48% of their previous under-performance in only 55 days of the re-alimentation period, this was determined through calculation of the CG index^2^, through comparison of growth rates between RES and ADLIB groups.

### Gene co-expression analysis

**Figure 1:**
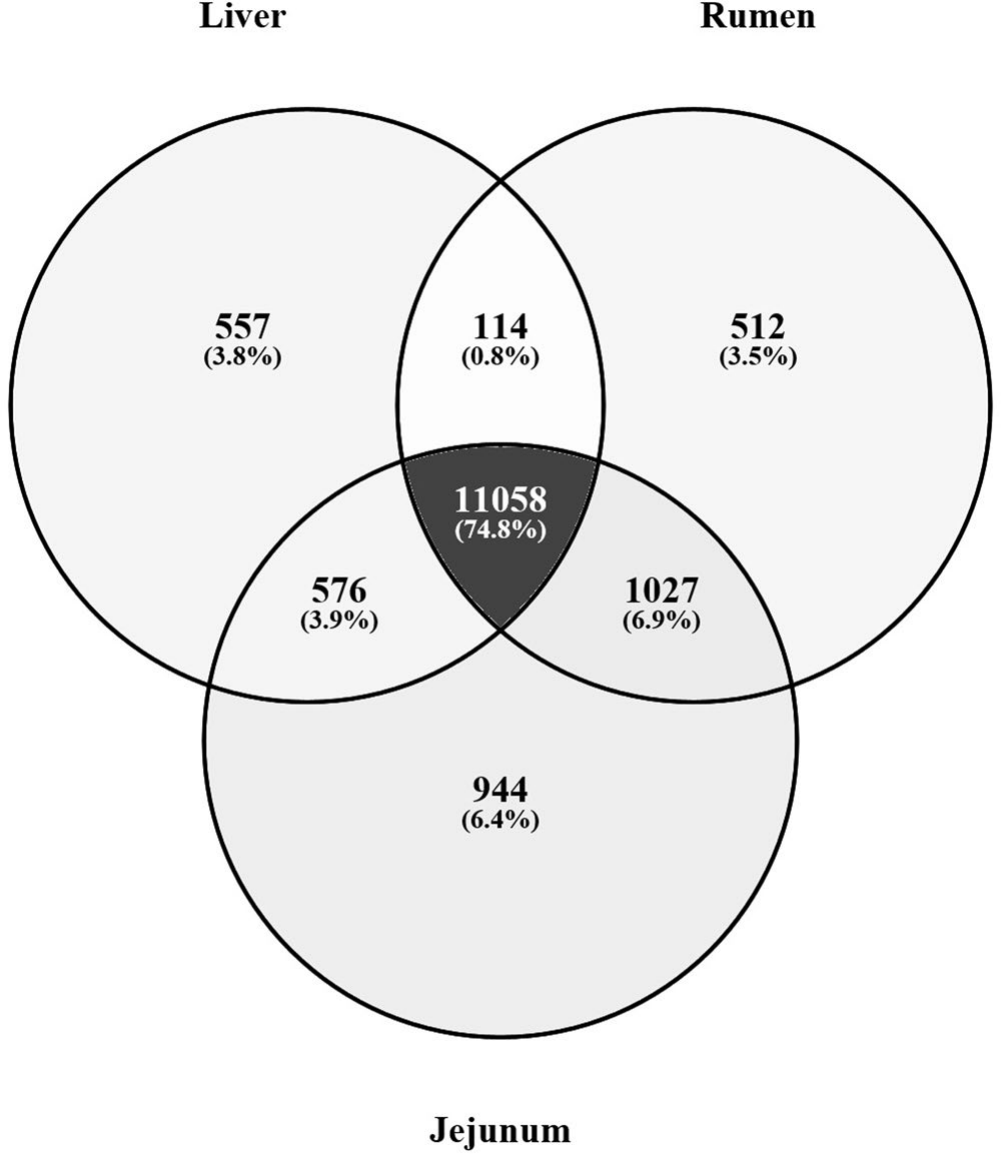
Venn diagram of genes commonly expressed between metabolic tissues in animals undergoing compensatory growth. Following alignment to the bovine genome, 12,305 genes were expressed in liver tissue, 12,711 genes were expressed in rumen papillae and 13,605 genes were expressed in jejunum. From the 3 tissues examined, a total of 11,058 genes or 74.8% of those expressed were commonly expressed across the differing tissue types, with these specific genes utilised for gene co-expression network analysis.

**Figure 2:**
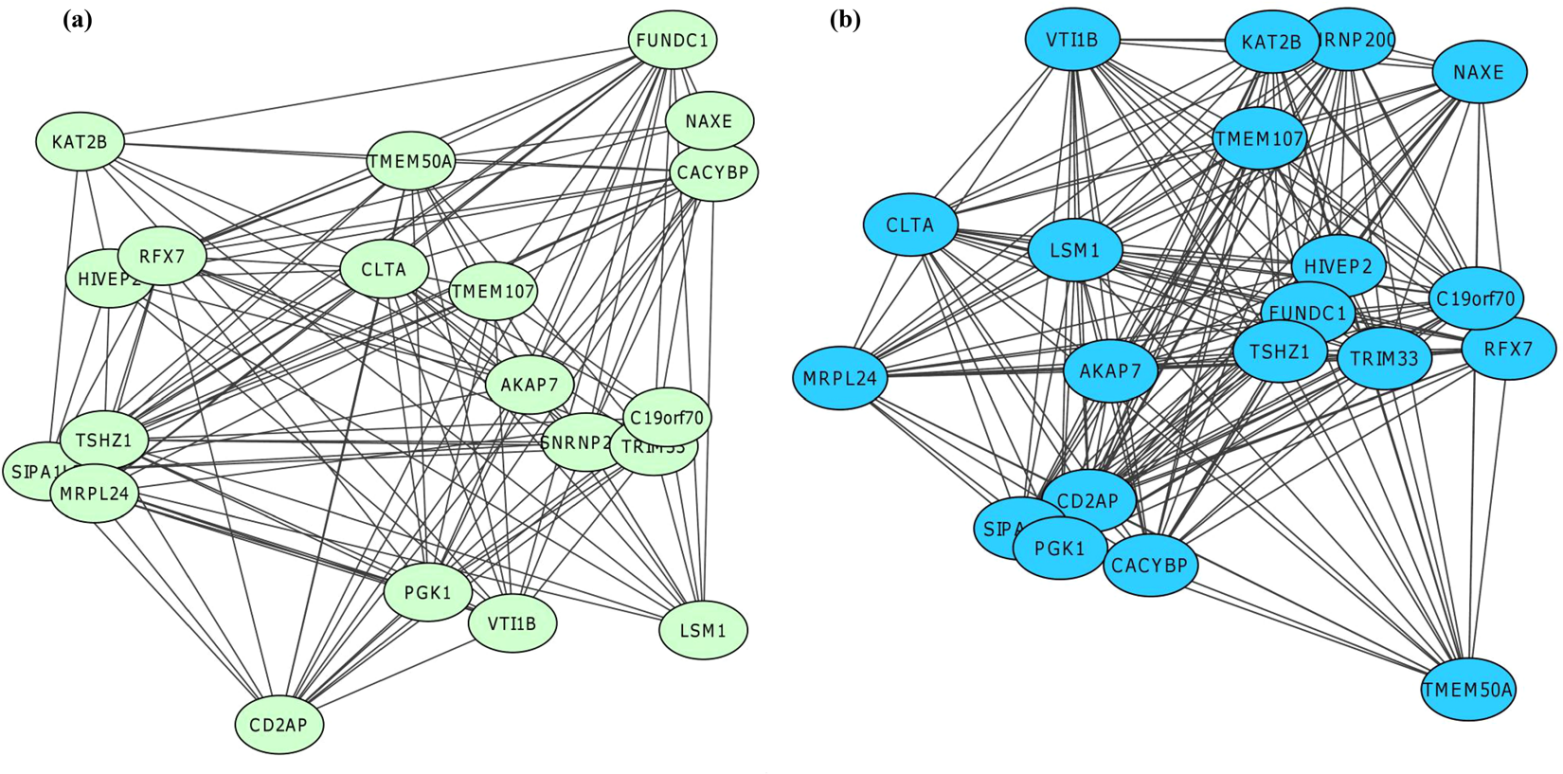
Top hub genes of the green module from (**a**) liver and (**b**) jejunum epithelium specific expression profiles. The green module of co-expressed genes was positively associated with feed conversion ratio across both liver and jejunum datasets, suggesting that hub genes within this module may hold potential for use as biomarkers for both improved compensatory growth and feed efficiency in cattle. The same hub genes were apparent across both tissue types, however the weight of the interaction (edge) between genes was different dependant on the tissue type.

Gene co-expression analysis was performed specifically on samples derived from cattle that had undergone CG (i.e. RES animals and not on ADLIB animals). Following alignment of RNAseq data to the bovine genome, 12,305 genes were expressed in liver tissue, 12,711 genes were expressed in rumen papillae and 13,605 genes were expressed in jejunum. From the 3 tissues examined, a total of 11,058 genes or 74.8% of the total list of genes expressed across the differing tissues were commonly expressed (Fig. 1). Weighted gene co-expression network analysis (WGCNA) was used to construct modules of genes that were co-expressed from the list of commonly expressed genes and WGCNA consensus analysis was subsequently performed to evaluate the similarity of gene expression profiles across the differing tissue types. A total of 15 modules or clusters of co-expressed genes were generated from the list of 11,058 commonly expressed genes, through WGCNA consensus analysis. In the liver dataset, 7 modules were significantly associated with production traits assessed; 2 modules were positively associated with ADG (magenta module: r = 0.67, P = 0.03; turquoise module: r = 0.66, P = 0.04), with 3 modules negatively associated with ADG (pink module: r = −0.69, P = 0.03; green module: r = −0.87, P = 0.0004; red module: r = −0.71, P = 0.02). Feed conversion ratio was also positively associated with the green module within the liver dataset (r = 0.8, P = 0.004). For the rumen dataset, 2 significant associations were identified between DMI per unit of bodyweight, namely the pink module (r = 0.69, P = 0.02) and the purple module (r = 0.65, P = 0.04). Within the jejunum dataset, 1 module of co-expressed genes (cyan module) was positively associated with DMI per unit of bodyweight (r = 0.72, P = 0.03) and FCR (r = 0.88, P = 0.0008), as well as negatively associated with ADG (r = −0.93, P = 0.0005). The green module of co-expressed genes was also positively associated with FCR in the jejunum dataset (r = 0.68, P = 0.04). Trait-module correlation results are presented in Supplementary File 1 (Fig. S1a–c for jejunal, ruminal and liver datasets, respectively). When co-expression profiles were evaluated across datasets, only 1 module of co-expressed genes was similar, the ‘green’ module was significantly positively correlated across jejunum and liver datasets (Fig. S2b; r = 0.68, P = 0.04). The top hub genes for this module across liver and jejunal datasets were visualised using cytoscape and are presented in Fig. 2a,b, respectively. Between the 2 datasets, although co-expressed gene results were similar across the 2 datasets, the interactions between genes were not the same, with edges between genes (reflecting the strength of the interaction between genes) different depending on the tissue type (Fig. 2). Consensus analysis results across datasets are presented in Supplementary File 1 (Fig. S2a–c). Multiple testing correction of p-values resulted in 2 module-trait relationships surviving significance with a further association tending towards significance following Benjamini-Hochberg correction. These included the green module with ADG in liver tissue (adj. P-value = 0.044) and cyan module with ADG in jejunal epithelium (adj. P-value = 0.013). Additionally the cyan module correlation with FCR in the jejunal epithelium tended towards significance (adj, P-value = 0.059). Due to current discussion within the literature in relation to the necessity in performing a multiple-testing correction of P-values derived from module-trait relationships of gene co-expression analyses^20,21^, both corrected and uncorrected P-values are presented and discussed within this manuscript. P-values and adjusted P-values for individual tissue module-trait relationships as well as consensus module-trait relationships are presented in Supplementary File 1 (Tables S1–6). Gene ontology analysis was performed on each module of co-expressed genes found to be significantly associated with production trait data (magenta, turquoise, pink, green, red, purple, cyan). Top ontology results for each of these modules of co-expressed genes are presented in Table 1. Genes within significantly associated modules of co-expressed genes identified in this study as well as previously identified as differentially expressed in the literature in relation to CG and feed efficiency in cattle in additional studies are presented in Table 2.

**Table 1:** Functional annotation of significant modules of co-expressed genes associated with compensatory growth.

Module	Gene ontology^a^	P value^b^
Magenta	Proteinaceous extracellular matrix, CC	1.20E-04
	Extracellular matrix structural constituent, MF	1.90E-06
	Collagen trimmer, CC	2.20E-06
	Collagen type IV trimmer, CC	2.20E-05
Turquoise	Chromatin, CC	7.30E-03
	Nucleotide binding, MF	2.40E-03
	Structural constituent of ribosome, MF	8.10E-03
	Translation, BP	4.50E-02
Pink	Translation, BP	4.40E-40
	Translational initiation, BP	1.90E-05
	Regulation of translational initiation, BP	2.10E-04
	Eukaryotic translation initiation factor 3 complex, CC	3.30E-04
	Formation of translation pre-initiation complex, BP	1.70E-03
	Mitochondrial translational elongation, BP	3.00E-04
	Mitochondrial translational initiation, BP	3.00E-04
	Mitochondrial large ribosomal subunit, CC	1.30E-03
Purple	Tricarboxylic acid cycle, BP	1.00E-04
	Mtiochondrial matrix, CC	3.40E-04
Cyan	Protein transport, BP	1.50E-02
	Transcription, DNA-templated, BP	2.60E-02
Green	Proteasome core complex, CC	7.80E-11
	Proteasome core complex, alpha-subunit complex, CC	1.50E-05
	Ubiquitin-dependent protein catabolic process, BP	3.30E-04
	Protein folding, BP	5.90E-04
	Proteasome accessory complex, CC	2.50E-03
	Proteasome regulatory particle, base subcomplex, CC	2.70E-02
	Ubiquitin protein ligase binding, MF	3.40E-03
	Ubiquitin protein ligase activity, MF	1.40E-02
Red	Mitochondrial respiratory chain complex I, CC	3.40E-02

^a^CC: cellular component; MF: molecular function; BP: biological process.

^b^P values have been corrected using Benjamini-Hochberg correction.

**Table 2:** Comparison of co-expressed genes from the current study with genes associated with compensatory growth and feed efficiency in the published literature.

Comparison	Common genes
Magenta module and Keogh *et al*.^7^ (liver)	*MMP14*
Magenta module and Connor *et al*.^26^	*ABCC9, ACADSB, CC2D2A, CCL16, COL15A1, COL4A1, COL4A5, COQ7, DHX40, ERP29, LRRC32, RGS5, RNASE4, SEC. 11* *C, SVEP1*
Turquoise module and Keogh *et al*.^7^ (liver)	*BCORL1*
Turquoise module and Connor *et al*.^26^	*ABLIM1, AKAP9, CALR, CLK1, CUL5, DCLRE1C, DLG1, DUSP6, EEA1, EIF5A, FRS2, GLG1, GOLGB1, H2AFY, ICMT, LMAN2, LSM10, MGA, MLH3, MLLT10, MRPS12, MYO5C, NKTR, NRIP1, PCF11, PFDN5, RBP1, RNPC3, RPL36A, RPS10, RPS15, RPS19, RSF1, SF3B1, SLC12A7, SLTM, SNX17, SSR2, UFC1, YLPM1, YTHDC2, ZNF638*
Green module with Tizioto *et al*.^38,45^, Chen *et al*.^30^, Weber *et al*.^34^, Kong *et al*.^28^, Zarek *et al*.^33^	*ATF3, BANF1, CCT5, PDRG1, PSMB5, PSMB6, PSMC5, S100A14, SEC. 61B, SNRNP40, TMEM147, TMSB10, TPI1, TUBB4B, WRB*

## Discussion

Compensatory growth is widely known to occur in ruminant species, particularly in cattle and consequently it is used in many production systems as a method to reduce feed costs^2,5,17,18^. Thus due to its common utilisation in beef cattle production systems, more effective utilisation of this naturally occurring phenomenon is of interest to animal producers internationally. One such means is through the identification of animals with a superior propensity to display CG, which could potentially be propagated in the cattle population through genomic selection breeding programs. We have previously performed gene expression studies aimed at identifying genes that were differentially expressed in animals undergoing CG compared to those that were fed continuously in tissues including liver, skeletal muscle and rumen and jejunal epithelium^6–9^. However such studies do not allow for an evaluation of all genes expressed in a tissue during a particular treatment nor do they provide information on the interaction of genes. Thus in the current study we sought to perform an evaluation of genes commonly expressed across tissues through gene co-expression analyses in an effort to identify key genes that may hold potential, following appropriate validation, for subsequent use as genetic biomarkers for the selection of cattle with a superior ability to display CG. Co-expression network analysis is a systems biology method for describing correlation patterns of genes across datasets, resulting in the formation of networks or clusters of highly correlated genes which may contribute to the expression of a particular trait^19^. These analyses provide a more holistic evaluation through the integration of gene expression data from multiple datasets as well as through incorporation of associated phenotypic data. This is achieved through examining all expressed genes as opposed to those detected as differentially expressed, allowing for a more equitable comparison across diverse study designs. Moreover, from networks of co-expressed genes, the most inter-connected genes, also known as hub genes, may be identified. Hub genes are genes that are important in regulating the expression of several other genes within a network and may be potential biomarkers for the selection of a trait. Such an approach has been utilised previously in ruminant studies for the identification of genes governing particular traits^22–25^, indeed we have previously evaluated gene co-expression patterns of jejunal epithelium in relation to CG in cattle^15^. Across the literature, metabolically active tissues have been shown to be most responsive to both dietary restriction and also to subsequent re-alimentation induced CG^16–18^. This has been reported through measurements of the size and weight of these organs^5,16^ as well as through alterations at the molecular level^6,8,9,26^. Thus we focused our attention towards genes commonly expressed in liver tissue and ruminal and jejunal epithelium datasets during CG.

The magenta module of co-expressed genes was significantly positively correlated with ADG in the liver dataset and with DMI (per kg bodyweight) in the rumen epithelium dataset. Gene ontology analysis of the co-expressed genes of this module revealed it to include genes involved in processes such as extracellular matrix collagen and collagen trimmer. As associations were positive between modules of co-expressed genes and DMI and ADG, this suggests greater expression of genes involved in these processes during enhanced DMI and ADG typically observed during CG^2,27^ and as reported in the animals used for this study^5^. A similar response was apparent for the turquoise module of co-expressed genes, where genes involved in processes including nucleotide binding, translation, the ribosome and chromatin were co-expressed and were also positively correlated with greater ADG rates in liver tissue of animals undergoing CG. These gene co-expression profiles may be due to a requirement for animals to build tissues during greater DMI while undergoing re-alimentation and may contribute to the greater rates of gain during CG through a necessity to deposit as much dietary energy as tissue should another period of dietary restriction re-occur. Alternatively greater expression of genes involved in extracellular matrix and collagen trimming may reflect an increase in cellular activity, as it has been suggested that with greater dietary intake there is a greater need for cellular activity to process the nutrients from the diet. A similar response has been reported in feed efficient cattle where Kong *et al*.^28^ and Chen *et al*.^29^ observed greater expression of genes involved in cellular assembly, organization and cytoskeletal dynamics in rumen epithelium and liver tissues, respectively, in animals with greater feed efficiency. Indeed this was evident in the skeletal muscle tissue of the animals used in the current study whereby following only 15 days of re-alimentation induced CG, greater expression of genes involved in cellular interactions and organization was apparent^7^. However it is worth noting that although such a response was apparent at day 15 of CG in muscle tissue, we did not identify such a response in any of the tissues used in this study under the previous differential expression analyses by day 55 of re-alimentation^6,8,9^. Indeed, genes within the magenta and turquoise modules of co-expressed genes were not necessarily identified as differentially expressed between animals undergoing CG and those on a continuous growth trajectory. For example upon comparison between the genes of the magenta module and differentially expressed genes in the liver based datasets of Keogh *et al*.^7^ and Connor *et al*.^26^ only 1 gene was common with our own previous differential gene expression dataset, *MMP14*, with 15 genes identified between the magenta module and the dataset of Connor *et al*.^26^. Within the turquoise module a similar finding was observed where although this module was found to contain 525 co-expressed genes, only 1 of these genes, *BCORL1*, had been identified as differentially expressed in our own liver differential expression based study^7^, and 42 genes were common with the differential expression dataset of Connor *et al*.^26^. When differential expression of liver tissue datasets of Keogh *et al*.^6^ and Connor *et al*.^26^ were directly compared, only 5 genes were identified as common between studies (*DDX58*, *SPARC*, *ABCG8*, *NANS*, *LYSMD3*). It is interesting to note that although only 5 genes were identified as commonly differentially expressed between these 2 liver based studies, when comparing the co-expressed genes from both the magenta and turquoise modules with the differential expression results of Connor *et al*.^26^ a larger number of common genes was apparent. The gene expression profiles of Connor *et al*.^26^ are reflective of the early stages of CG, with liver tissue samples harvested on day 1 and 14 of re-alimentation induced CG in that study, whereas our own liver based differential gene expression analysis was performed on day 55 of re-alimentation. Consequently common genes identified through gene co-expression analysis may represent regulatory genes, which may ultimately contribute to CG during different stages of re-alimentation and hence warrant further evaluation for use as DNA-based biomarkers for the selection of enhanced CG ability in cattle.

Enhanced mitochondrial energy production has been implicated as contributing to CG^7,26^ as well as in improved feed efficiency state^30^ in cattle. However results to date on this are not conclusive. For example data from our own group showed that during CG lower expression of oxidative phosphorylation genes was evident in skeletal muscle tissue following 15 days of re-alimentation induced CG^7^. Conversely though, Connor *et al*.^26^ reported greater expression of mitochondrial genes during the first days of re-alimentation following a previous phase of dietary restriction. The differences between these studies, as previously stated, may have been attributed to differences in tissue sampling, with the work of Connor *et al*.^26^ focusing on the very early part of re-alimentation. However the potential lack of enhanced mitochondrial energy efficiency towards CG is further established in the current study, through negative associations of the pink and red modules with ADG. Co-expressed genes within the pink module reflected those involved in mitochondrial translation, with those of the red module involved in mitochondrial respiratory chain complex. However, although this difference between studies may be attributable to differences in sampling time-points, there may be tissue specific differences in relation to mitochondrial functionality during CG. This is evident through the positive correlation between the pink module of co-expressed genes and the greater DMI observed during re-alimentation in the ruminal epithelium dataset, suggesting that mitochondrial efficiency may contribute to CG in a tissue dependent manner. Moreover, a positive correlation was also evident for the purple module of co-expressed genes in the rumen epithelium and DMI, with the purple module involved in the tricarboxylic acid cycle as well as the mitochondrial matrix. In their rumen epithelium based study, Kong *et al*.^28^ reported a negative association between a module of co-expressed genes involved in oxidative phosphorylation and greater feed efficiency (through low-Residual Feed Intake), further implicating enhanced mitochondrial energy production within the rumen. Greater DMI has been reported during CG^5,27,31^, with an increased appetite thought to be contributing to the expression of CG in cattle^5^. Additionally the rumen has been shown to be one of the most responsive tissues to dietary restriction and also subsequent re-alimentation^5^, with fluctuations in the weight of this organ directly proportional to dietary intake^32^. These results suggest that CG of rumen tissue in cattle may be a consequence of a change in feed intake, which is not unexpected given the metabolic role of the tissue as well as its direct response to feeding level^32^. Overall these results suggest a potential differential regulation of mitochondrial efficiency dependent on not only the duration of CG and re-alimentation but also on the tissue type. However functional analysis of mitochondrial respiration rate during different stages of CG as well as in different tissues is warranted to fully elucidate the contribution of mitochondrial energy production to CG.

During CG cattle have repeatedly been shown to be more feed efficient^2,5^, indeed the animals used in the current study displayed a better (numerically lower) FCR during re-alimentation^5^. Within the jejunum dataset the cyan module of co-expressed genes was found to be positively correlated with FCR. This module of co-expressed genes codes for proteins involved in processes including protein transport and transcription. Greater expression of genes coding for these processes has previously been reported in beef cattle with greater feed efficiency state in comparison to their inefficient counterparts^29,33^. Indeed 2 genes in particular from the cyan module of co-expressed genes, C4orf32 and *MIER1* were also identified as contributing to residual feed intake in cattle through gene co-expression analysis in the work of Weber *et al*.^34^ and Kong *et al*.^28^ respectively. C4orf32 is a protein that functions in the endoplasmic reticulum^35^ and *MIER1* codes for a transcriptional repressor involved in chromatin silencing^36^. However although results from these studies implicate these genes as potentially important for improved feed efficiency in cattle, the results of the current study do not agree with this. This was apparent through the positive association between the cyan module containing *MERI1* and C4orf32 and FCR suggesting that greater expression of these genes was associated with a less favorable FCR. Additionally the negative association between this module of co-expressed genes and ADG suggests that the expression of these genes was lower in animals displaying greater daily growth rates. Moreover another gene within this module, *EXTL2* was previously identified as up-regulated in low feed-efficiency Jersey cows compared to their high-efficiency counterparts^37^. Consequently further evaluations of these particular genes are warranted in order to truly assess their potential use as molecular biomarkers for cattle with improved feed efficiency status.

From the current analysis, only 1 module of co-expressed genes was identified through WGCNA consensus analysis as similar across datasets, namely the green module was similarly positively correlated with FCR across jejunum and liver datasets. This module of co-expressed genes was also significantly associated with ADG within the liver dataset. The green module, comprised of 316 co-expressed genes, is involved in functions including proteasome, protein folding, proteolysis and ubiquitination. However although the correlation was positive, as improved FCR is numerically lower, this suggests that the expression values of these genes were in fact lower in our datasets during CG. Additionally, the negative association between this module within the liver dataset and ADG suggests that the expression of these genes were in fact lower in animals displaying greater ADG values during CG. Furthermore when compared against available feed efficiency datasets, 16 genes from this module had been previously reported in relation to differential expression between animals divergent for feed efficiency phenotype (Table 2). Moreover, of these 16 genes only 1 gene of the green module of co-expressed genes had been identified in multiple feed efficiency datasets, namely the *TMSB10* gene which was previously identified as differentially expressed in liver and rumen epithelium datasets of Tizioto *et al*.^38^ and Kong *et al*.^28^, respectively. Additionally, in both studies expression was greater in the feed efficient animals compared to their inefficient counterparts. The implication of this gene across multiple datasets and experimental settings may imply its utility as a potential biomarker for the selection of animals with superior feed efficiency potential. The *TMSB10* gene plays an important role in the organization of the cytoskeleton and can inhibit actin polymerization^39^. Similarly a role for cytoskeletal organization was previously described in both skeletal muscle and ruminal epithelium tissues of cattle undergoing CG^7,9^, emphasizing the importance of this function to the expression of greater feed efficiency potential during CG.

Top hub genes for the green module of co-expressed genes have not been reported as differentially expressed in previous CG gene expression studies, nor were these genes implicated in feed efficiency investigations. Lack of commonality between top hub genes of this module and genes identified as differentially expressed in relation to CG and feed efficiency in the literature may be due to timing of sampling e.g. in early CG in Connor *et al*.^26^. However these genes may be implicated towards an improved feed efficiency state during CG in the current study due to evaluating all expressed genes through co-expression network analysis and evaluating overall regulation of the expression of CG in cattle. Upon further evaluation, top hub genes were comprised of genes involved in functions such as cellular interactions and cellular adhesion (*ADAM19*, *CD93*, *FAT4*, *LUM*, *SRPX*, *TMEM17*), cellular signaling (*ARHGAP29*, *DGKA*, *GNB4*, *RGS3*, *FZD1*), gene expression and protein synthesis (*EAPP*, *MRPS27*, *PGPEP1*) and proliferation and growth (*BMP4*, *FLT4*, *PDGFRB*). Each of these processes have previously been implicated in terms of CG in cattle, however hub genes have not previously been identified as differentially expressed in those studies^6–9,26^. A similar finding was evident in our evaluation of the liver dataset alone^40^, where the top hub genes of a module positively associated with DMI had not been identified as differentially expressed in the differential gene expression study^6^, further establishing the utility of gene co-expression analyses and importance of knowledge of gene interactions in terms of identifying specific genes and biochemical pathways important to particular traits of interest. Additionally, hub genes in the green module of co-expressed genes in the current study which was positively correlated with FCR and thus feed efficiency were also not previously identified as differentially expressed in the literature. Furthermore, as these genes are key hub genes modulating the expression of other genes in this module, this suggests that they may represent potential biomarkers for the selection of cattle with a greater feed efficiency potential. However further evaluations are warranted to prove the efficacy of these genes as biomarkers for CG in cattle.

The data presented in this study builds upon previous results in the literature in relation to genes contributing to CG through differential gene expression analysis and our preliminary gene co-expression analysis of the jejunal transcriptome. A period of CG resulted in modules of co-expressed genes across metabolically active tissues with both positive and negative associations evident with ADG, FCR and DMI. Co-expressed genes of significant modules included those involved in processes including gene expression, mitochondrial respiratory chain, and extracellular matrix. One module of co-expressed genes was found to be similar across the jejunum and liver gene expression datasets. This module included genes involved in proteasome core complex, signal transduction and protein synthesis. Results from this study showed that a large portion of co-expressed genes in significant modules had not previously been identified in studies examining CG through differential gene expression, thus this study identifies novel genes involved in controlling the expression of CG, with hub genes holding potential for use as biomarkers for the selection of animals with a greater propensity to display CG. However, although this study provides new information of the genetic regulation of CG in cattle, it is limited due to the inclusion of datasets from only 1 set of animals. An evaluation of gene expression datasets from animals under different experimental environments may lead to the identification of robust biomarkers for the selection of cattle with improved ability to display CG. Additionally, although the tissue samples and data used for this study were derived from a highly controlled experimental environment, it is necessary for this study to be replicated with larger population sizes and in independent samples for validation of the results presented but also for the identification of reliable molecular biomarkers for CG in cattle. Nevertheless, hub genes identified in this study warrant further attention for their potential use for the selection of cattle with an enhanced ability to display CG. A further evaluation of any potential variants at the DNA level of these genes between animals divergent in CG potential will add to this investigation.

## Methods

The University College Dublin Animal Research Ethics Committee approved all procedure using animals and the current study was licensed by the Irish Department of Health and Children in accordance with the European Community Directive 86/609/EC.

### Animal management

Tissue samples used in this study were derived from a large animal model experiment aimed at describing the effect of both dietary restriction and subsequent re-alimentation induced CG on overall body physiology^5,41^. Specific details describing the management of animals used are outlined in detail in Keogh *et al*.^5,41^ and are only briefly described here. Thirty Holstein Friesian yearling bulls were separated into 2 dietary treatment groups; RES and ADLIB, with 15 animals in each. For 125 days (Period 1) RES animals were offered a restricted diet designed for animals to grow at 0.6 kg/day, following which they were offered *ad libitum* access to feed for a further 55 days (Period 2). The ADLIB group were offered *ad libitum* access to feed for the entire 180 days of the trial. All animals received the same diet throughout the trial but with different proportions based on treatment, and all animals were individually fed according to each animals own individual bodyweight.

### Tissue sampling

At the end of the 180 day trial all animals were slaughtered in an EU licensed abattoir (Euro Farm Foods Ltd, Cooksgrove, Duleek, Co. Meath, Ireland) through captive bolt stunning followed by exsanguination. Jejunal and ruminal epithelium and liver tissues were collected from all animals within 30 minutes of slaughter. In all cases samples were washed in Dulbecco’s phosphate buffered saline, and subsequently snap frozen in liquid nitrogen. Samples were then stored at −80 °C pending further processing. Tissue specific collection methods are outlined in Keogh *et al*.^6,8,9^ for liver, jejunal epithelium and ruminal epithelium, respectively.

### RNAseq analysis

RNA isolation and quality verification, cDNA library preparation, sequencing as well as bioinformatic analyses are described in detail in Keogh *et al*.^6,8,9^. Briefly, total RNA was isolated from tissue samples using the RNeasy Mini Kit (Qiagen, UK), according to the manufacturer’s instructions. cDNA libraries were prepared from 3 μg of high quality total RNA using the Illumina TruSeq RNA sample prep kit following the manufacturer’s instructions (Illumina, San Diego, CA, USA). cDNA libraries were multiplexed according to their respective sample specific adapters and 100 bp single end sequencing was performed on an Illumina HiSeq. 2000 sequencer. Following sequencing, resultant reads were first checked for quality using Fastqc software (version 0.10.0; http://www.bioinformatics.babraham.ac.uk/projects/fastqc/) and low quality reads were removed. Sequencing reads were aligned to the bovine reference genome (UMD3.1) using TopHat (version v2.0.9; https://ccb.jhu.edu/software/tophat/index.shtml) and aligned reads were then counted using HTSeq^42^. RNAseq datasets are available through NCBI’s Gene Expression Omnibus (GEO) through accession numbers GSE64285, GSE94004 and GSE89162 for liver, jejunual epithelium and ruminal epithelium respectively.

### Gene co-expression analysis

#### Gene co-expression consensus analysis

The WGCNA software package^19^ was used to identify modules of co-expressed genes. Although RNAseq data was available for both RES and ADLIB groups following periods of both dietary restriction (Period 1) and re-alimentation (Period 2), the primary focus of this study was to determine genes contributing to CG, consequently WGCNA was performed specifically on data pertaining to animals that had undergone CG only (i.e. RES group during Period 2). Data for ADLIB animals during Period 2 were not included as these animals had undergone a normal continuous growth trajectory and consequently were not comparable to the RES Period 2 animals, however these animals did serve to show that RES animals displayed CG, as outlined in the results section. In order to perform WGCNA, RNAseq read count data from each tissue were first filtered for lowly expressed genes and normalised in EdgeR by read counts per million mapped reads, (adjusted for sample library size). Normalised count data were then Log_2_(x + 1) transformed in R. Networks of co-expressed genes were constructed using WGCNA within R, utilising the available online tutorials and manuals (https://horvath.genetics.ucla.edu/html/CoexpressionNetwork/Rpackages/WGCNA/Tutorials/). Unsigned, weighted correlation network construction and module detection were performed using the automatic one-step function, blockwiseModules consensus analysis, modules of co-expressed genes were based on genes commonly expressed across the 3 tissues (11058 genes; Fig. 1). Between all pairs of genes for each tissue, pair-wise weighted Pearson correlations were calculated. In order to reach scale free topology of the network (R^2^ > 0.9), an adjacency matrix was calculated by raising the co-expression matrix to a soft-threshold power. The soft-thresholding powers used were as follows: 10 for jejunal epithelium, 18 for liver and 19 for rumen epithelium. Based on the transformed connection strengths between genes a topology overlap matrix was calculated, providing biologically meaningful information of the similarity of the co-expression between 2 genes with all other genes in the network. Average linkage hierarchical clustering was then applied to the topology overlay matrix resulting in the grouping of highly similarly expressed genes and modules of co-expressed genes subsequently identified by the dynamic tree cut algorithm. Modules with highly correlated eigengens were then merged, with modules containing at least 30 co-expressed genes assigned a colour by the software.

#### Module-trait relationships

Modules of co-expressed genes were then correlated with trait production data. In the current study, trait data included traits that are associated with CG including greater ADG, improved FCR and greater DMI (per unit of bodyweight). The module eigengene is defined as the first principal component of a given module and provides a representative measure of gene expression profiles in a module. In order to determine the association between modules of co-expressed genes and phenotype data, Module-Trait relationships were calculated by Pearson correlation between the eigengene of each module and the specific phenotype data. Modules with statistically significant (P < 0.05) correlations were selected for further analysis as potentially biologically interesting modules associated with the expression of CG. Additionally, module-trait relationship p-values were also corrected for multiple testing by using the Benjamini-Hochberg method.

#### Hub gene identification and visualization

To identify key genes within a module, the eigengene based connectivity kME was used as a measure of module membership. Genes with a high module membership may be referred to as intramodular hub genes. These genes are representative for the entire module and are most likely key biological players in the respective module^14^. To visualize the network of top hub genes, the 30 genes with the highest module membership (nodes) were extracted and the strongest gene-gene connections (edges) from the topology overlay matrix. Cytoscape software^43^ (version 3.4.0) was used to visualize the networks using node and edge files as input for this analysis.

#### Gene Ontology

Gene ontology analysis was subsequently performed on genes from each module identified through consensus analysis as significantly correlated with trait data (P < 0.05). The Database for Annotation, Visualization and Integrated Discovery (DAVID)^44^ was used for functional annotation of co-expressed genes within modules. The entire list of all genes in significant modules was used as a custom background for gene ontology analysis. To correct for multiple testing of gene ontology analysis, P-values were adjusted using Benjamini-Hochberg correction. Gene ontology terms were considered significant if the adjusted Benjamini-Hochberg corrected P-value was less than 0.05.

## Supplementary information

Gene co-expression networks contributing to the expression of compensatory growth in metabolically active tissues in cattle Supplemetary information

## Data Availability

The datasets analysed during the current study are available in the NCBI Gene Expression Omnibus https://www.ncbi.nlm.nih.gov/geo/ under accession numbers GSE64285, GSE94004 and GSE89162.
