# Supplementary material for: Gene co-expression networks contributing to the expression of compensatory growth in metabolically active tissues in cattle
Source: Sci Rep. 2019 Apr 15;9:6093. doi: 10.1038/s41598-019-42608-w (PMC6465245; doi:10.1038/s41598-019-42608-w)
Supplement: Supplementary file 1 — Gene co-expression networks contributing to the expression of compensatory growth in metabolically active tissues in cattle Supplemetary information [file 41598_2019_42608_MOESM1_ESM.pdf]

Supplementary information

Gene co-expression networks contributing to the expression of compensatory growth in metabolically active tissues in cattle

Authors: Kate Keogh, David A. Kenny and Sinead M. Waters*

Animal and Bioscience Research Department, Animal and Grassland Research and Innovation Centre, Teagasc, Grange, Dunsany, Co. Meath, Ireland

*Corresponding author:

E-mail: Sinead.Waters@teagasc.ie

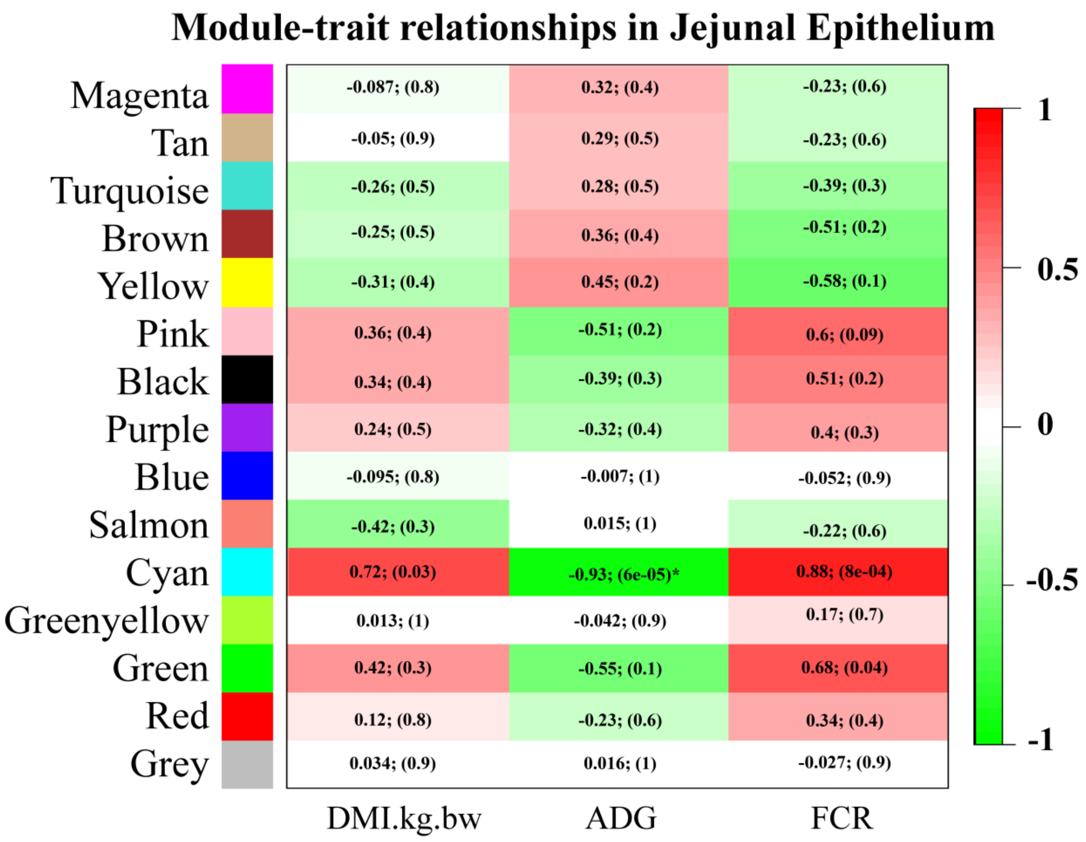

Figure S1a. Correlation results between modules of co-expressed genes (denoted by colours on left side) and trait data including DMI (dry matter intake), ADG (average daily gain) and FCR (feed conversion ratio) in jejunal epithelium of animals undergoing compensatory growth. Correlation coefficients are presented followed by P values (uncorrected) in parenthesis. *Indicates module that passed multiple-testing correcting (adj. P-value = 0.013).

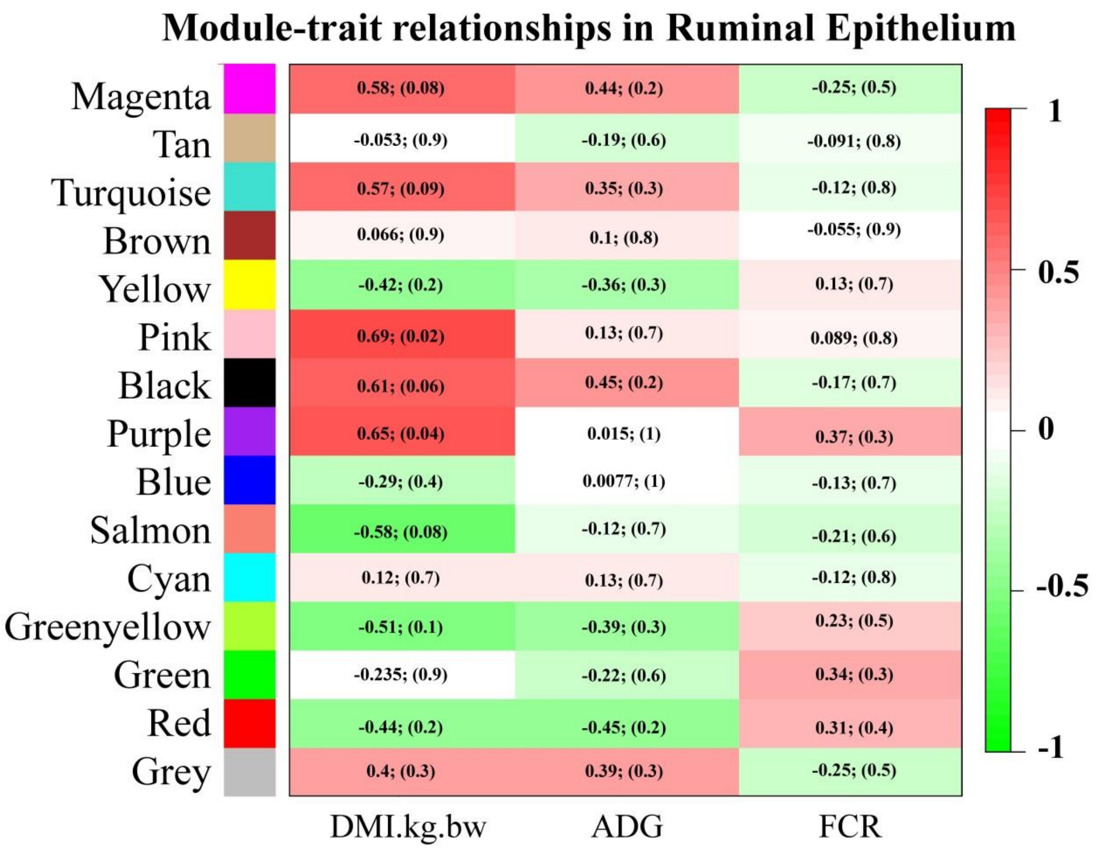

Figure S1b. Correlation results between modules of co-expressed genes (denoted by colours on left side) and trait data including DMI (dry matter intake), ADG (average daily gain) and FCR (feed conversion ratio) in ruminal epithelium of animals undergoing compensatory growth. Correlation coefficients are presented followed by P values (uncorrected) in parenthesis.

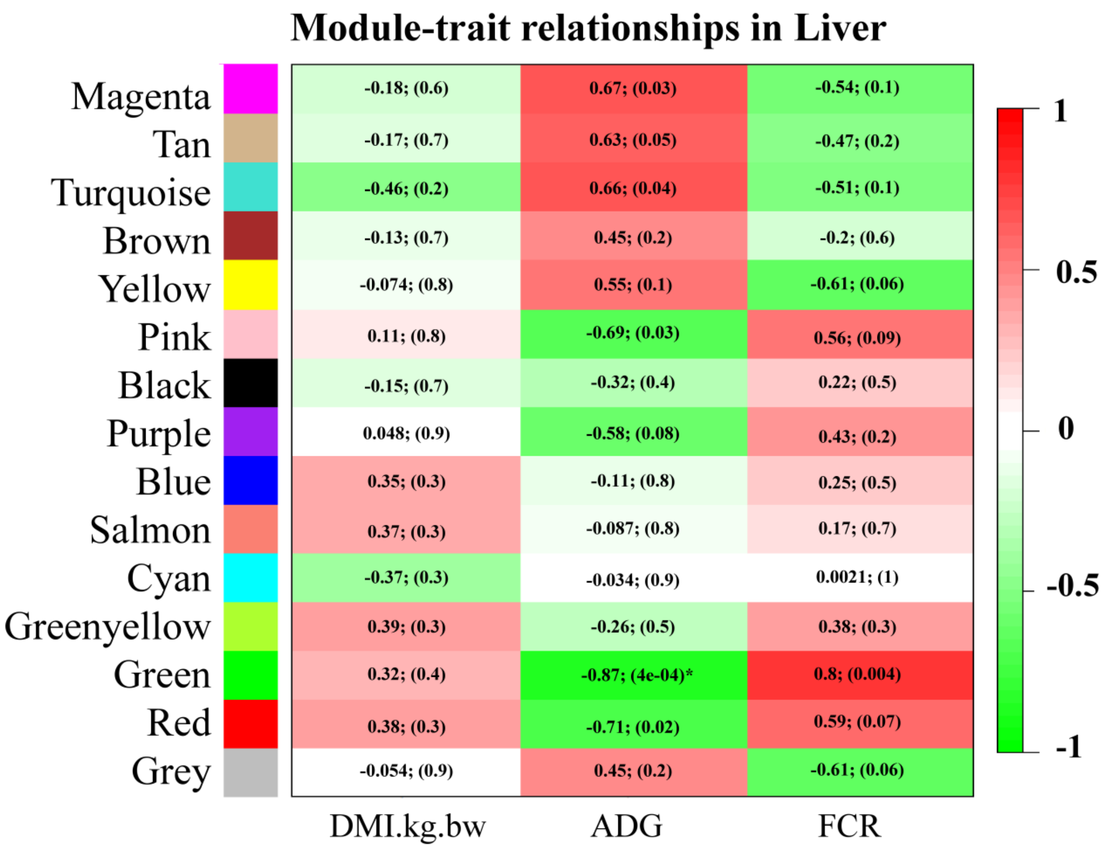

Figure S1c. Correlation results between modules of co-expressed genes (denoted by colours on left side) and trait data including DMI (dry matter intake), ADG (average daily gain) and FCR (feed conversion ratio) in hepatic tissue of animals undergoing compensatory growth. Correlation coefficients are presented followed by P values (uncorrected) in parenthesis. *Indicates module that passed multiple-testing correcting (adj. P-value = 0.044).

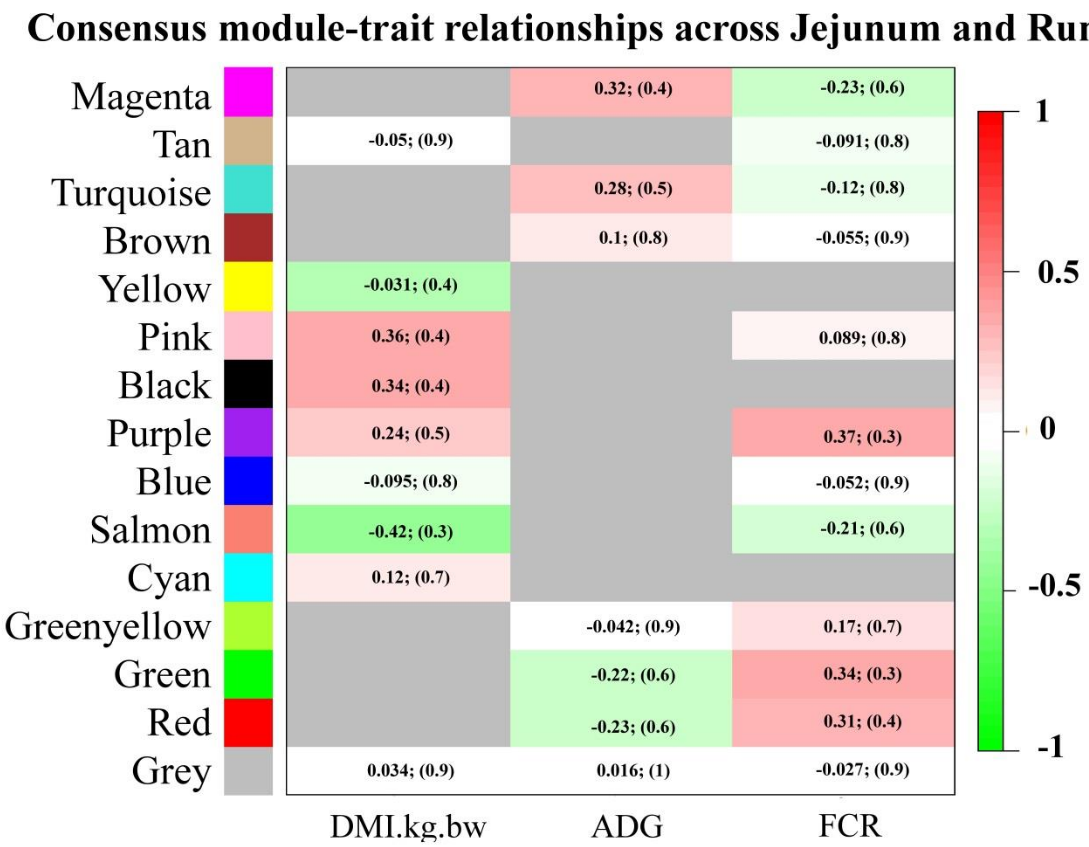

Figure S2a. Consensus analysis between jejunum and rumen datasets between correlation results of modules of co-expressed genes (denoted by colours on left side) and trait data including DMI (dry matter intake), ADG (average daily gain) and FCR (feed conversion ratio) in hepatic tissue of animals undergoing compensatory growth. Correlation coefficients are presented followed by P values (uncorrected) in parenthesis. Grey boxes indicate that the correlations between the two tissue types have opposite signs and consequently no consensus can be formed.

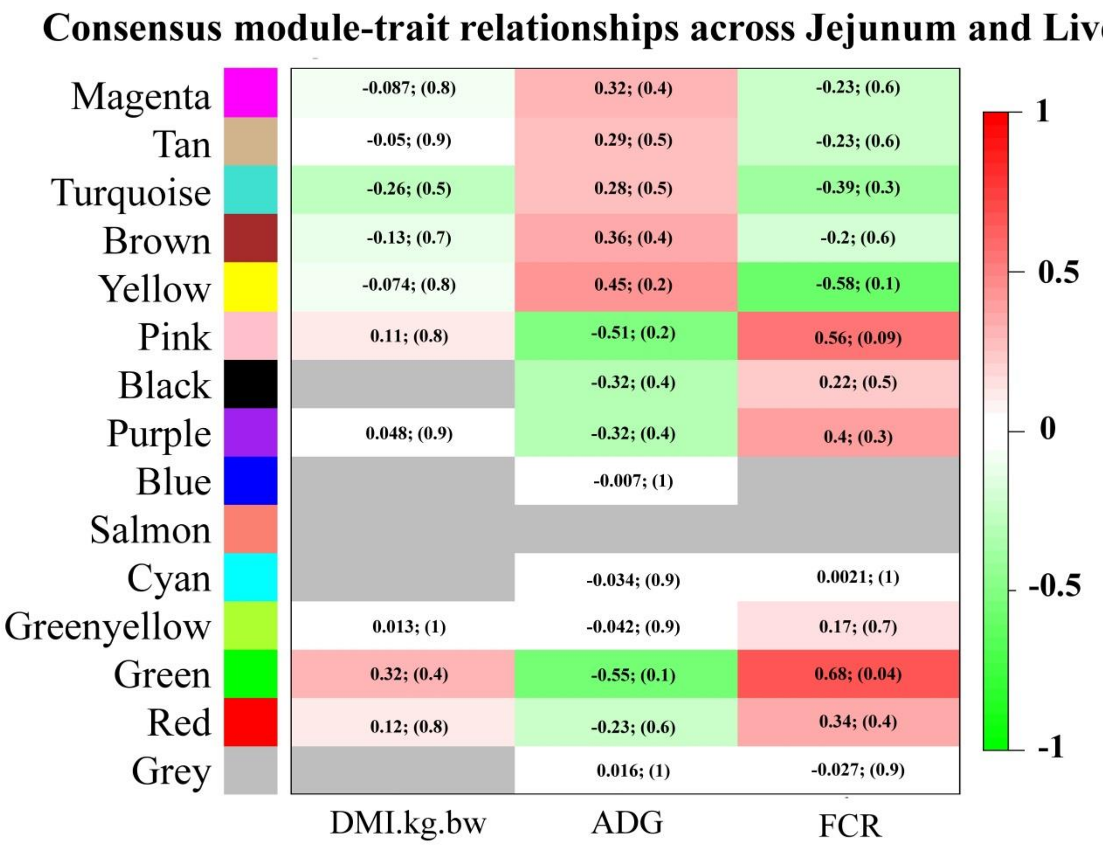

Figure S2b. Consensus analysis between jejunum and liver datasets between correlation results of modules of co-expressed genes (denoted by colours on left side) and trait data including DMI (dry matter intake), ADG (average daily gain) and FCR (feed conversion ratio) in hepatic tissue of animals undergoing compensatory growth. Correlation coefficients are presented followed by P values (uncorrected) in parenthesis. Grey boxes indicate that the correlations between the two tissue types have opposite signs and consequently no consensus can be formed.

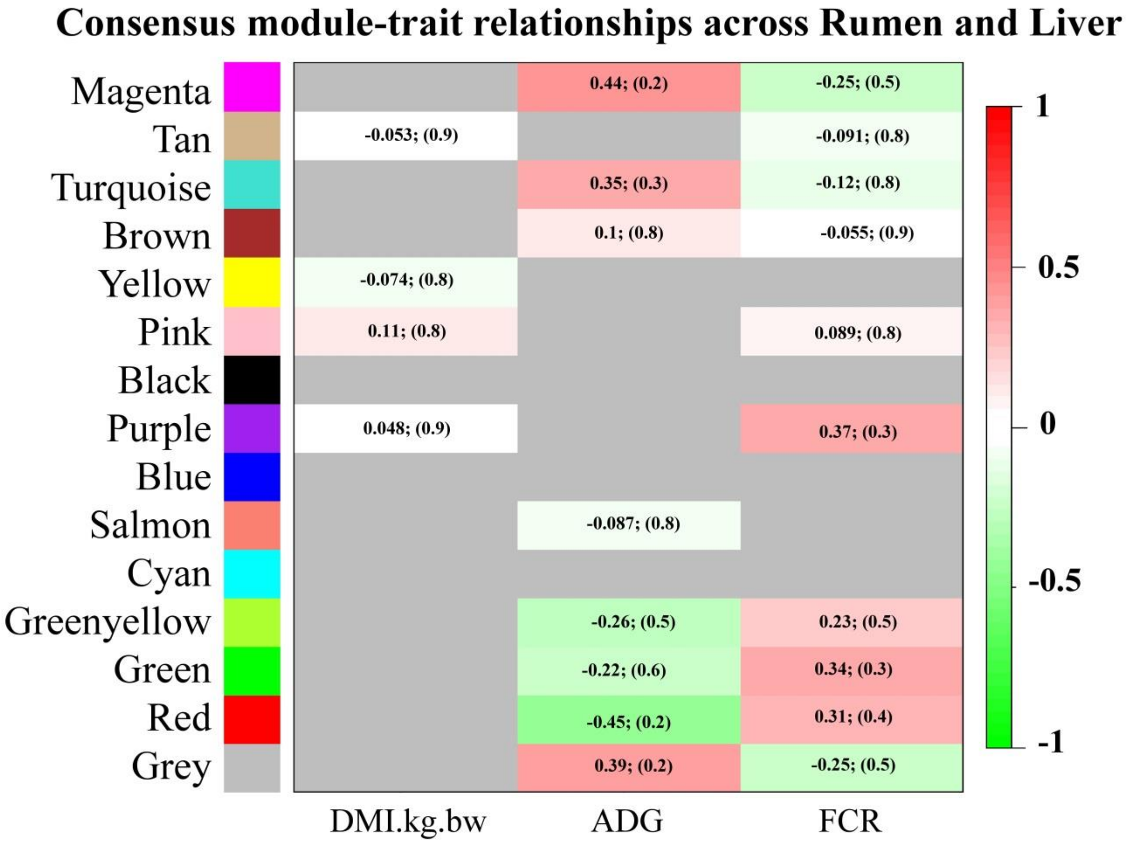

Figure S2c. Consensus analysis between rumen and liver datasets between correlation results of modules of co-expressed genes (denoted by colours on left side) and trait data including DMI (dry matter intake), ADG (average daily gain) and FCR (feed conversion ratio) in hepatic tissue of animals undergoing compensatory growth. Correlation coefficients are presented followed by P values (uncorrected) in parenthesis. Grey boxes indicate that the correlations between the two tissue types have opposite signs and consequently no consensus can be formed.

Supplementary Table S1. P-value and adjusted P-value (adj. P-value) for module trait relationships in jejunal epithelium.

	DMI[1]		ADG[2]		FCR[3]	
Module	P-value	adj. P-value	P-value	adj. P-value	P-value	adj. P-value
Magenta	0.8	0.951	0.4	0.879	0.6	0.951
Tan	0.9	0.951	0.5	0.910	0.6	0.951
Turquoise	0.5	0.910	0.5	0.910	0.3	0.854
Brown	0.5	0.910	0.4	0.879	0.2	0.854
Yellow	0.4	0.879	0.2	0.854	0.1	0.673
Pink	0.4	0.879	0.2	0.854	0.09	0.673
Black	0.4	0.879	0.3	0.854	0.2	0.854
Purple	0.5	0.910	0.4	0.879	0.3	0.854
Blue	0.8	0.951	1	1.000	0.9	0.951
Salmon	0.3	0.854	1	1.000	0.6	0.951
Cyan	0.03	0.673	6.00E-05	0.013	8.00E-04	0.059
Greenyellow	1	1.000	0.9	0.951	0.7	0.951
Green	0.3	0.854	0.1	0.673	0.04	0.673
Red	0.8	0.951	0.6	0.951	0.4	0.879
Grey	0.9	0.951	1	1.000	0.9	0.951

[1] Dry matter intake

[2] Average daily gain

[3] Feed conversion ratio

Supplementary Table S2. P-value and adjusted P-value (adj. P-value) for module trait relationships in ruminal epithelium.

	DMI[1]		ADG[2]		FCR[3]	
Module	P-value	adj. P-value	P-value	adj. P-value	P-value	adj. P-value
Magenta	0.08	0.673	0.2	0.854	0.5	0.910
Tan	0.9	0.951	0.6	0.951	0.8	0.951
Turquoise	0.09	0.673	0.3	0.854	0.8	0.951
Brown	0.9	0.951	0.8	0.951	0.9	0.951
Yellow	0.2	0.854	0.3	0.854	0.7	0.951
Pink	0.02	0.673	0.7	0.951	0.8	0.951
Black	0.06	0.673	0.2	0.854	0.7	0.951
Purple	0.04	0.673	1	1.000	0.3	0.854
Blue	0.4	0.879	1	1.000	0.7	0.951
Salmon	0.08	0.673	0.7	0.951	0.6	0.951
Cyan	0.7	0.951	0.7	0.951	0.8	0.951
Greenyellow	0.1	0.673	0.3	0.854	0.5	0.910
Green	0.9	0.951	0.6	0.951	0.3	0.854
Red	0.2	0.854	0.2	0.854	0.4	0.879
Grey	0.3	0.854	0.3	0.854	0.5	0.910

[1] Dry matter intake

[2] Average daily gain

[3] Feed conversion ratio

Supplementary Table S3. P-value and adjusted P-value (adj. P-value) for module trait relationships in liver.

	DMI[1]		ADG[2]		FCR[3]	
Module	P-value	adj. P-value	P-value	adj. P-value	P-value	adj. P-value
Magenta	0.6	0.951	0.03	0.673	0.1	0.673
Tan	0.7	0.951	0.05	0.673	0.2	0.854
Turquoise	0.2	0.854	0.04	0.673	0.1	0.673
Brown	0.7	0.951	0.2	0.854	0.6	0.951
Yellow	0.8	0.951	0.1	0.673	0.06	0.673
Pink	0.8	0.951	0.03	0.673	0.09	0.673
Black	0.7	0.951	0.4	0.879	0.5	0.910
Purple	0.9	0.951	0.08	0.673	0.2	0.854
Blue	0.3	0.854	0.8	0.951	0.5	0.910
Salmon	0.3	0.854	0.8	0.951	0.7	0.951
Cyan	0.3	0.854	0.9	0.951	1	1.000
Greenyellow	0.3	0.854	0.5	0.910	0.3	0.854
Green	0.4	0.879	4.00E-04	0.044	0.004	0.222
Red	0.3	0.854	0.02	0.673	0.07	0.673
Grey	0.9	0.951	0.2	0.854	0.06	0.673

[1] Dry matter intake

[2] Average daily gain

[3] Feed conversion ratio

Supplementary Table S4. P-value and adjusted P-value (adj. P-value) for consensus module trait relationships across jejunum and rumen epithelium.

	DMI[1]		ADG[2]		FCR[3]	
Module	P-value	adj. P-value	P-value	adj. P-value	P-value	adj. P-value
Magenta			0.4	0.879	0.6	0.951
Tan	0.9	0.951			0.8	0.951
Turquoise			0.5	0.910	0.8	0.951
Brown			0.8	0.951	0.9	0.951
Yellow	0.4	0.879				
Pink	0.4	0.879			0.8	0.951
Black	0.4	0.879				
Purple	0.5	0.910			0.3	0.854
Blue	0.8	0.951			0.9	0.951
Salmon	0.3	0.854			0.6	0.951
Cyan	0.7	0.951				
Greenyellow			0.9	0.951	0.7	0.951
Green			0.6	0.951	0.3	0.854
Red			0.6	0.951	0.4	0.879
Grey	0.9	0.951	1	1	0.9	0.951

[1] Dry matter intake

[2] Average daily gain

[3] Feed conversion ratio

Supplementary Table S5. P-value and adjusted P-value (adj. P-value) for consensus module trait relationships across jejunum epithelium and liver.

	DMI[1]		ADG[2]		FCR[3]	
Module	P-value	adj. P-value	P-value	adj. P-value	P-value	adj. P-value
Magenta	0.8	0.951	0.4	0.879	0.6	0.951
Tan	0.9	0.951	0.5	0.910	0.6	0.951
Turquoise	0.5	0.910	0.5	0.910	0.3	0.854
Brown	0.7	0.951	0.4	0.879	0.6	0.951
Yellow	0.8	0.951	0.2	0.854	0.1	0.673
Pink	0.8	0.951	0.2	0.854	0.09	0.673
Black			0.4	0.879	0.5	0.910
Purple	0.9	0.951	0.4	0.879	0.3	0.854
Blue			1	1.000		
Salmon						
Cyan			0.9	0.951	1	1
Greenyellow	1	1.000	0.9	0.951	0.7	0.951
Green	0.4	0.879	0.1	0.673	0.04	0.673
Red	0.8	0.951	0.6	0.951	0.4	0.879
Grey			1	1	0.9	0.951

[1] Dry matter intake

[2] Average daily gain

[3] Feed conversion ratio

Supplementary Table S6. P-value and adjusted P-value (adj. P-value) for consensus module trait relationships across rumen epithelium and liver.

	DMI[1]		ADG[2]		FCR[3]	
Module	P-value	adj. P-value	P-value	adj. P-value	P-value	adj. P-value
Magenta			0.2	0.854	0.5	0.910
Tan	0.9	0.951			0.8	0.951
Turquoise			0.3	0.854	0.8	0.951
Brown			0.8	0.951	0.9	0.951
Yellow	0.8	0.951				
Pink	0.8	0.951			0.8	0.951
Black						
Purple	0.9	0.951			0.3	0.854
Blue						
Salmon			0.8	0.951		
Cyan						
Greenyellow			0.5	0.910	0.5	0.910
Green			0.6	0.951	0.3	0.854
Red			0.2	0.854	0.4	0.879
Grey			0.2	0.854	0.5	0.910

[1] Dry matter intake

[2] Average daily gain

[3] Feed conversion ratio
